# Mina-Brasil – a birth cohort to study health and nutrition in the Amazon

**DOI:** 10.11606/s1518-8787.2023057supl2ed

**Published:** 2024-02-01

**Affiliations:** I Universidade Federal de São Paulo Escola Paulista de Medicina São Paulo SP Brasil Universidade Federal de São Paulo., Escola Paulista de Medicina. São Paulo, SP, Brasil; II Universidade de São Paulo Faculdade de Saúde Pública Departamento de Nutrição São Paulo SP Brasil Universidade de São Paulo. Faculdade de Saúde Pública. Departamento de Nutrição. São Paulo, SP, Brasil

The challenges to promote health, already complex due to their multidimensional nature, are magnified by the diversity of realities and inequities that characterize Brazil, a country with exceptionally particular scenarios in its needs and opportunities.

Knowledge production contributes to the management of public actions and policies by shedding light on these specificities. Thus, the construction of interinstitutional partnerships configures a strategy that, among other obvious benefits, contributes to shortening the distances of this continental country and offers a promising path to better apply research resources and train teams under a collaborative networking perspective.

A team of researchers from Universidade São Paulo, Universidade Federal do Acre (at its Floresta campus in CZS), and Harvard University (Boston, USA) has followed a cohort of children and their mothers living in the municipality of Cruzeiro do Sul (CZS), in inner Acre, since 2015. The São Paulo Research Foundation (FAPESP) and the National Council for Scientific and Technological Development (CNPq) support the Mina-Brasil Study – Maternal and Child Health and Nutrition in Acre. This is the first population-based birth cohort in the Western Brazilian Amazon^1^. The region is known for its high malaria transmission: about 90% of all Brazilian cases. In 2016, CZS, with less than 100,000 inhabitants, had the fourth highest annual incidence of malaria in Brazil, estimated at 231.9 cases per thousand inhabitants^2^.

This supplement of *Revista de Saúde Pública* gathered five studies that analyzed data obtained during the first five years of life of children participating in the Mina-Brasil cohort. Results show particular aspects of this group's health and nutrition conditions. Findings include the low frequency of exclusive breastfeeding and the occurrence of depressive symptoms among mothers, which point to the need for strengthening pre- and perinatal support, especially in the group exposed to greater social vulnerability. From 2015 to 2016, only 37% of the children in the Mina-Brasil study were exclusively breastfed (EBF) until their first month of life. At six months, this prevalence decreased to 10%, despite the World Health Organization recommending EBF up to this age^3^.

The prospective monitoring of growth from birth to five years of age showed that, although mean weight and height resulted in nutritional status indicators close to reference medians, malnutrition and overweight coexisted in the study population. The dynamics of nutritional status in these children points to the low probability of the spontaneous reversal of adversely affected growth patterns, especially during pregnancy and in the first two years of life^4^.

Moreover, two other studies deal with the application of a research instrument to track child behavior problems in the Amazon and the trajectories of depressive symptoms in mothers, with consequences for their children's mental health^5,6^.

Finally, results on nutritional status up to the fifth year of life provide an overview of the epidemiologically relevant nutritional deficiencies, such as anemia. Iron deficiency (ID) remains very common, affecting 43% of local pregnant women and 38% of children aged one year but its contribution to anemia seems to vary with age as 51% of pregnant women and 62% of one-year-old children had anemia and iron deficiency, whereas 32% of anemic children at two years of age had ID. In the first year of life, in addition to ID, the consumption of ultra-processed foods was also associated with the risk of anemia. Vitamin A deficiency and insufficiency were also common during pregnancy (6% and 20%, respectively) and in two-year-old children (25% and 42%, respectively), but substantially less frequent in the first year of life (2% and 10%, respectively) when childhood anemia was more prevalent. Vitamin A status and malaria contributed to persistent anemia at two years of age in this population.^7^

*Revista de Saúde Pública* restates its commitment to scientific communication associated with surveillance actions by publishing part of the data from the Mina-Brasil Study in this supplement, which will certainly contribute to a better understanding of the phenomena, continuity of research, and loco-regional management of health actions.

Enjoy your reading!
