# Supplementary material for: Mina-Brasil – a birth cohort to study health and nutrition in the Amazon
Source: Rev Saude Publica. 2024 Feb 1;57(Suppl 2):1s. doi: 10.11606/s1518-8787.2023057supl2ed (PMC10897960; doi:10.11606/s1518-8787.2023057supl2ed)
Supplement: Translation

# Translation: MINA-Brasil – uma coorte de nascimentos para estudar a saúde e a nutrição na Amazônia

**Affiliations:** I São Paulo SP Brasil Universidade Federal de São Paulo. , Escola Paulista de Medicina. São Paulo, SP, Brasil; II São Paulo SP Brasil Universidade de São Paulo. Faculdade de Saúde Pública. Departamento de Nutrição. São Paulo, SP, Brasil

Os desafios para a promoção da saúde, já complexos por sua natureza multidimensional, são agigantados pela diversidade de realidades e inequidades que caracterizam o Brasil, um país com cenários excepcionalmente particulares em suas necessidades e oportunidades.

A produção de conhecimento contribui para a gestão das ações e políticas públicas, ao trazer clareza sobre essas especificidades. Neste sentido, a construção de parcerias interinstitucionais é uma estratégia que, entre outros benefícios evidentes, contribui para o encurtamento das distâncias deste país de dimensões continentais. Este é também um caminho promissor para a melhor aplicação de recursos de pesquisa, e para a qualificação de equipes em uma perspectiva de redes colaborativas.

Desde 2015, uma coorte de crianças e suas mães residentes na cidade de Cruzeiro do Sul (CZS), interior do Acre, vem sendo acompanhada por uma equipe de pesquisadores da Universidade de São Paulo, da Universidade Federal do Acre no campus Floresta em CZS e da Universidade de Harvard (Boston, EUA); o Estudo Mina-Brasil - Saúde e Nutrição Materno-Infantil no Acre, tem apoio da Fundação de Amparo à Pesquisa do Estado de São Paulo (FAPESP) e do Conselho Nacional de Desenvolvimento Científico e Tecnológico (CNPq). Trata-se da primeira coorte de nascimentos de base populacional na Amazônia Ocidental Brasileira^1^. A região é reconhecida pela alta transmissão da malária: cerca de 90% dos casos do país ocorrem nessa região. Em 2016, CZS com menos de 100.000 habitantes apresentou a quarta maior incidência anual de malária no Brasil, estimada em 231,9 casos por mil habitantes^2^.

Neste suplemento da Revista de Saúde Pública reunimos 5 artigos com análise de dados obtidos ao longo dos 5 primeiros anos de vida das crianças participantes da coorte MINA-Brasil. Os resultados apresentados revelam aspectos particulares das condições de saúde e nutrição deste grupo; entre os achados, a baixa frequência de aleitamento exclusivo e a ocorrência de sintomas depressivos entre as mães, apontam para a necessidade de fortalecimento do apoio pré e peri-natal, especialmente no grupo exposto a maior vulnerabilidade social. Entre 2015 e 2016, apenas 37% das crianças do estudo MINA-Brasil foram amamentadas exclusivamente (AME) até o primeiro mês de vida. Aos 6 meses, a prevalência de aleitamento materno exclusivo diminuiu para 10%, apesar da recomendação da Organização Mundial da Saúde preconizar AME até os 6 meses de idade^3^.

O monitoramento prospectivo do crescimento a partir do nascimento até os 5 anos de idade mostrou que, a despeito do peso e a estatura médias resultarem em indicadores de estado nutricional próximos às medianas de referência, desnutrição e sobrepeso coexistem na população do estudo. A dinâmica do estado nutricional observada entre as crianças aponta para a baixa probabilidade de reversão espontânea de padrões de crescimento adversamente afetados, especialmente durante a gestação e nos dois primeiros anos de vida^4^.

Outros dois artigos tratam da aplicação de instrumento de pesquisa para o rastreamento de problemas de comportamento infantil no contexto Amazônico, e das trajetórias de sintomas depressivos nas mães, com desdobramentos sobre a saúde mental de seus filhos^5,6^.

Finalmente, resultados sobre o estado nutricional até o quinto ano de vida desenham um panorama sobre carências nutricionais de relevância epidemiológica, como a anemia. A deficiência de ferro (DF) continua muito comum, afetando 43% das mulheres grávidas locais e 38% das crianças com 1 ano de idade, mas a sua contribuição para a anemia parece variar com a idade: DF foi observada em 51% das mulheres grávidas e 62% das crianças de 1 ano de idade que estavam anêmicas, enquanto 32% das crianças anêmicas aos 2 anos de idade apresentaram DF. No primeiro ano de vida, além da DF, o consumo de alimentos ultraprocessados também foi associado ao risco para anemia. Deficiência e insuficiência de vitamina A também foram comuns durante a gravidez (6% e 20%, respectivamente) e em crianças de 2 anos (25% e 42%, respectivamente), mas substancialmente menos frequentes no primeiro ano de vida (2% e 10%, respectivamente), quando a anemia infantil foi mais prevalente. O estado de vitamina A e a malária contribuíram para a anemia persistente aos dois anos de vida nessa população^7^.

A Revista de Saúde Pública reafirma seu compromisso com a comunicação científica associada às ações de vigilância ao publicar, neste suplemento, parte dos dados do Estudo Mina-Brasil, que certamente contribuirão para a melhor compreensão dos fenômenos, para a continuidade da pesquisa, e para a gestão loco-regional das ações em saúde.

Boa leitura!
